# Geographical Distribution, Spatial Directional Trends, and Spatio-Temporal Clusters of the First Rapid and Widespread Lumpy Skin Disease Outbreaks in Thailand

**DOI:** 10.1155/tbed/4900775

**Published:** 2025-02-24

**Authors:** Kusnul Yuli Maulana, Kannika Na-Lampang, Orapun Arjkumpa, Noppawan Buamithup, Kannikar Intawong, Veerasak Punyapornwithaya

**Affiliations:** ^1^Faculty of Veterinary Medicine, Chiang Mai University, Chiang Mai, Thailand; ^2^Research Center for Veterinary Biosciences and Veterinary Public Health, Faculty of Veterinary Medicine, Chiang Mai University, Chiang Mai, Thailand; ^3^Animal Health Section, The 4th Regional Livestock Office, Department of Livestock Development, Khon Kaen, Thailand; ^4^Department of Livestock Development, Ministry of Agriculture and Cooperatives, Bangkok, Thailand; ^5^Faculty of Public Health, Chiang Mai University, Chiang Mai, Thailand

## Abstract

Thailand was recognized as having the highest number of lumpy skin disease (LSD) outbreaks in Southeast Asia during 2021. Understanding how LSD outbreaks spread over time and space can provide detailed insight into the distribution and pattern of the disease, allowing for more precise identification of areas with high disease burden. This study aims to explore the spread of LSD among cattle in Thailand during 2021 using spatial and spatio-temporal analyses. Data were analyzed using spatial analysis techniques, including spatial autocorrelation and directional distribution. Additionally, the spatio-temporal models, including space–time permutation (STP) and Poisson with various maximum reported cluster size (MRCS) settings, were applied to the data to determine LSD outbreak clusters. Results showed that a total of 642 LSD outbreaks were reported from March to December 2021. Districts with confirmed cases exhibited spatial autocorrelation, indicating the interconnected spread of LSD across different geographic areas. Furthermore, the disease distribution pattern appeared to extend to the southern and southwestern regions from the northeast. Based on the spatio-temporal models, LSD outbreak clusters were identified in several regions. The STP model tended to identify more clusters with smaller radii compared to the Poisson model. The number of clusters detected varied according to both the model and MRCS setting, underscoring the importance of selecting the most relevant clusters for the effective implementation of disease control strategies. This study was the first of its kind to assess the spatial direction and spatio-temporal distribution of LSD outbreak clusters based on national-level data. Evaluating LSD occurrence through spatial and spatio-temporal analyses can provide valuable insight into its spatio-temporal dynamics, facilitating disease surveillance, control measures, and vector control strategies in Thailand.

## 1. Introduction

Lumpy skin disease (LSD) is a vector-borne disease caused by the lumpy skin disease virus (LSDV), which is regarded as one of the most harmful animal pox viruses because of its detrimental effects on the cattle industry [1]. LSD is therefore classified as a notifiable disease criterion by the World Organisation for Animal Health (WOAH) due to its significant economic impact [2]. In 2019, LSD outbreaks were reported in Bangladesh, India, and China, with Bangladesh identified as the first hotspot in South Asia [3, 4]. In Southeast Asia, LSD outbreak was first reported from Vietnam in 2020 [5]. Subsequently, the first case of LSD in Thailand was reported in March 2021 in the northeastern part of Thailand, with further cases continuing to be reported [6–8]. In 2021, according to WOAH, Thailand had the highest number of reported LSD outbreaks among Southeast Asian countries [9]. The number of reported LSD outbreaks in Asia increased by about threefold each year from 2019 to 2021 [10].

Spatial analysis techniques are essential for monitoring, preventing, and controlling livestock diseases [11]. The analysis produced data on the geographic spread of diseases, thus pinpointing regions with elevated disease burdens that require urgent intervention through control measures [12]. Various spatial analysis methods can be employed to examine the outbreak and generate valuable insights, including spatial autocorrelation analysis through Moran's I, standard deviation ellipse (SDE), and spatio-temporal analysis [13].

Spatial autocorrelation analysis has been extensively employed in the field of epidemiology [14]. For instance, it has been utilized in the study of various diseases such as dengue [15], tuberculosis [16], and coronavirus (COVID-19) [17]. In animals, this approach is also widely used in Bovine tuberculosis [18], foot and mouth disease [19], and Trypanosomiasis [20]. This analytical approach serves to investigate the geographical patterns inherent in disease spread, as well as the interconnectedness of occurrence rates among neighboring regions [21]. The spatial autocorrelation can be assessed using Moran's global autocorrelation index and Moran's local index [22].

The SDE is a statistical method for examining and depicting directional patterns within spatial data [23]. It creates a new feature consisting of elliptical polygons centered on the mean center [24]. The versatility of SDE is evident across a spectrum of research disciplines, including tracking toxin dispersion in groundwater wells [25], analyzing household activities or travel behavior [26], and studying crime distribution trends [27]. For example, this technique has also been employed to investigate the spatial attributes of animal diseases, such as central tendencies, dispersion, and orientation trends, exemplified by studies on African swine fever in Poland [28] and the distribution pattern of LSD in Southeast Asia [10]. The orientation of the elliptical polygon indicates the clockwise rotation of the longer axis [29], which helps in understanding the spatial expansion and directional tendencies of the point locations under scrutiny [28].

Spatial and spatio-temporal analyses are increasingly being used to investigate the disease burden by determining high-risk locations, temporal trends, and the factors leading to outbreaks [30]. Spatial clustering analysis has gained prominence across diverse research disciplines, notably within epidemiology [31]. In recent research, spatio-temporal analysis has been utilized to identify clusters and evaluate the risk factors associated with LSD in multiple regions, including countries in the Asian continent [32], India [33], and Uganda [34]. Previous investigations have also examined the spatio-temporal clustering of LSD outbreaks in Thailand, employing various models [8, 35]. However, the limitation of these studies is that only locally collected LSD outbreak data are analyzed.

The selection of an appropriate spatio-temporal model in practical applications depends on factors such as data characteristics, research objectives, specific assumptions, and constraints of the model [36]. The default maximum spatial window size (MSWS) parameter, typically set at 50%, covers 50% of the population at risk [37, 38]. However, the MSWS approach varies in some research publications, as outlined in the previous study [39]. Unfortunately, this approach has some issues based on statistical concepts [40]. The SatScan User Guide indicates that conducting the analysis multiple times with different MSWS values may introduce potential multiple testing issues [39]. Therefore, it is suggested that the original default MSWS value be analyzed while adjusting the maximum reported cluster size (MRCS) values [40, 41]. The concept and mathematical formula regarding the variation of MRCS are thoroughly explained in a previous study [40].

LSD outbreaks were first reported across Thailand in 2021, negatively impacting cattle production. Since LSD is relatively new to Thailand and currently under surveillance, gaining a deeper understanding of LSD epidemiology is essential. While publications on LSD epidemiology exist in Thailand, none have explored the spatial epidemiology of LSD at a national scale, resulting in knowledge gaps on this aspect. Therefore, this study aims to investigate the spatial epidemiology and spatio-temporal clusters of LSD outbreaks in Thailand. The findings from this study will provide both global and localized insights into the distribution and patterns of LSD outbreaks in Thailand, enhancing the understanding of LSD epidemiology and its associated burden.

## 2. Materials and Methods

### 2.1. Data and Data Management

Data on LSD outbreaks in Thailand compiled by the authors at the Department of Livestock Development (DLD), Thailand, was used in this study. Furthermore, these data were submitted to WOAH, making them accessible through the World Animal Health Information System (WAHIS) at https://wahis.woah.org. The data covered the period from 10 March 2021 to 31 December 2021, consisting of the onset date of the outbreak, geographic coordinates (latitude/longitude), unit classification (village or farm), and counts of susceptible animals and cases. The data were collected from all provinces experiencing LSD outbreaks. In the LSD outbreak reporting system, livestock authorities at the provincial level collected daily data from districts. Subsequently, they validated and electronically transferred the data to the central DLD. Data were further managed and validated by the central DLD authority. In this study, a total of 642 LSD outbreak reports were utilized for further analyses.

### 2.2. Mapping

For the choropleth map, the districts of Thailand were used as observation units to assess the dissemination of LSD. Thailand encompasses a total of 928 districts, constituting the primary geographical entities under scrutiny. All data were stored and managed as a geographical database with QGIS v3.34.2-Prizren (Free Software Foundation, Boston, USA).

A choropleth map was generated utilizing the Jenks natural breaks classification method conducted in GeoDa software v1.22.0.4 (GeoDa Center) to stratify data according to inherent groupings within the dataset. Within this map representation, a quantitative attribute is displayed per spatial unit, presenting ordinal classes of LSD outbreak occurrence in 642 districts from a total of 928 districts in Thailand. Generally, for a choropleth map, the shading of areas corresponds to their respective occurrence values, with a spectrum of shading classes employed. Darker shades denote higher disease occurrence, while lighter shades indicate lower occurrence levels.

### 2.3. Data Analysis

#### 2.3.1. Spatial Autocorrelation Analysis

In this study, GeoDa was utilized to examine the spatial autocorrelation of LSD outbreaks based on the global Moran's I and the local Moran's I indices within the local indicators of spatial association (LISA) to investigate the global and localized spatial correlation patterns, respectively [21].

The global and local Moran's I tests were performed using a first-order Queen's contiguity spatial weights matrix, incorporating data from all adjacent regions to evaluate whether a region demonstrates a higher or lower mean, thereby gauging the extent of spatial autocorrelation [42]. Moran's I is defined as follows [43]:

(1) $$\begin{array}{c} I=\frac{n\left(\sum_{i=1}^{n}\sum_{j=1}^{n}w_{ij}\left(x_{i}-\overset{―}{x}\right)\left(x_{j}-\overset{―}{x}\right)\right)}{\left(\sum_{i=1}^{n}\sum_{j=1}^{n}w_{ij}\right)\left(\sum_{i=1}^{n}{\left(x_{i}-\overset{―}{x}\right)}^{2}\right)}. \end{array}$$

In the formula, *n* refers to the number of districts involved in the analysis. The terms *x*_*i*_ and *x*_*j*_ represent the observational values of cases *x* on the *i* and *j* of the space unit where *w*_*ij*_ is the spatial weight. Moran's I statistics range between (−1 and 1). A value closer to 1 signifies stronger positive spatial autocorrelation, while a value closer to (−1) suggests stronger negative spatial autocorrelation.

Furthermore, LISA was employed to deal with insight into the local clusters of LSD outbreaks. Four discernible categories of local spatial autocorrelation emerge between a given regional unit and its adjacent area units. These categories include instances where both the regional unit and its adjacent area units exhibit high values (H–H), both demonstrate low values (L–L), the regional unit features a high value while its adjacent area units display low values (H–L), the regional unit has a low value while its adjacent area units exhibit high values (L–H) [22]. These distinctions in local spatial connectivity patterns provide insight into the relational dynamics between neighboring geographical units.

#### 2.3.2. Directional Distribution Based on SDE

The SDE was employed to examine point patterns, offering a synopsis of the outbreak distribution in the form of an ellipse. The analysis was conducted using the QGIS SDE plugin. The two methods currently available in the plugin, namely the Yuill and CrimeStat, were used in this study.

The SDE was used to delineate LSD outbreaks, accounting for size and encompassing one standard deviation of the *x* coordinates and *y* coordinates from the mean center throughout the study period. The SDE is defined as follows [44]:

(2) $$\begin{array}{c} {\text{SDE}}_{x}=\frac{\sum_{i=1}^{n}{\left(x_{i}-\overset{―}{X}\right)}^{2}}{n} \end{array}$$

(3) $$\begin{array}{c} {\text{SDE}}_{y}=\sqrt{\frac{\sum_{i=1}^{n}{\left(y_{i}-\overset{―}{Y}\right)}^{2}}{n}}, \end{array}$$

where *x*_*i*_ and *y*_*i*_ are the coordinates for feature *i*, {$\overset{―}{X}$, $\overset{―}{Y}$} represents the mean center for the features, and *n* is the sum of the features.

The angle of rotation is computed as follows [44]:

(4) $$\begin{array}{c} \tan\;\theta =\frac{A+B}{C} \end{array}$$

(5) $$\begin{array}{c} A=\left(\sum_{i=1}^{n}\;\overset{―}{x_{i}^{2}}-\sum_{i=1}^{n}\;\overset{―}{y_{i}^{2}}\right) \end{array}$$

(6) $$\begin{array}{c} B=\sqrt{{\left(\sum_{i=1}^{n}\overset{―}{x_{i}^{2}}-\sum_{i=1}^{n}\overset{―}{y_{i}^{2}}\right)}^{2}+4{\left(\sum_{i=1}^{n}{\overset{―}{x}}_{i}{\overset{―}{y}}_{i}\right)}^{2}} \end{array}$$

(7) $$\begin{array}{c} C=2\sum_{i=1}^{n}{\overset{―}{x}}_{i}{\overset{―}{y}}_{i}, \end{array}$$

where $\overset{―}{x}$_*i*_ and $\overset{―}{y}$_*i*_ are the deviations of the *x* and *y* coordinates from the mean center.

The standard deviations for the *x*-axis and *y*-axis are [44]:

(8) $$\begin{array}{c} \sigma_{x}=\sqrt{2}\sqrt{\frac{\sum_{i=1}^{n}{\left({\overset{―}{x}}_{i}\cos\theta -{\overset{―}{y}}_{i}\sin\theta \right)}^{2}}{n}} \end{array}$$

(9) $$\begin{array}{c} \sigma_{y}=\sqrt{2}\sqrt{\frac{\sum_{i=1}^{n}{\left({\overset{―}{x}}_{i}\sin\theta -{\overset{―}{y}}_{i}\cos\theta \right)}^{2}}{n}} \end{array}$$

The angle of rotation indicates the orientation of the ellipse, representing the clockwise rotation of its longitudinal axis from the noon position [45].

#### 2.3.3. Spatio-Temporal Models

The spatial scan statistics in the SaTScan software v10.1.2 (https://www.satscan.org/) were used to identify disease clusters [46]. This statistical approach aims to discern regions exhibiting elevated or diminished disease risk by systematically scanning a window across the study area. The spatio-temporal scan statistic operates via a dynamic cylindrical window characterized by a circular geographic base and a height corresponding to time.

The space–time permutation (STP) and Poisson models were employed to determine clusters of LSD outbreaks. The STP model utilizes case data alongside geographic coordinates and the onset date of the outbreak. In contrast, the Poisson model incorporates additional factors such as the number of cases, overall animal count in each unit, unit coordinates, and the onset date of outbreaks [46]. The identification of the most likely cluster area was achieved through the maximum likelihood ratio. Furthermore, Monte Carlo simulation methodology was applied to ascertain the significance of candidate clusters, employing a total of 999 replications. Clusters exhibiting a *p*-value of less than 0.05 were construed as being statistically significant. The identified statistically significant clusters were mapped using QGIS v3.34.2-Prizren.

In the parameter setting, various MRCS values, 5%, 10%, 15%, 30%, and 50%, were employed for both the STP and Poisson models. This approach facilitated the identification of LSD outbreak clusters at both regional and local scales, aligning with the surveillance program in Thailand.

## 3. Results

### 3.1. Mapping of Outbreak Locations

**Figure 1:**
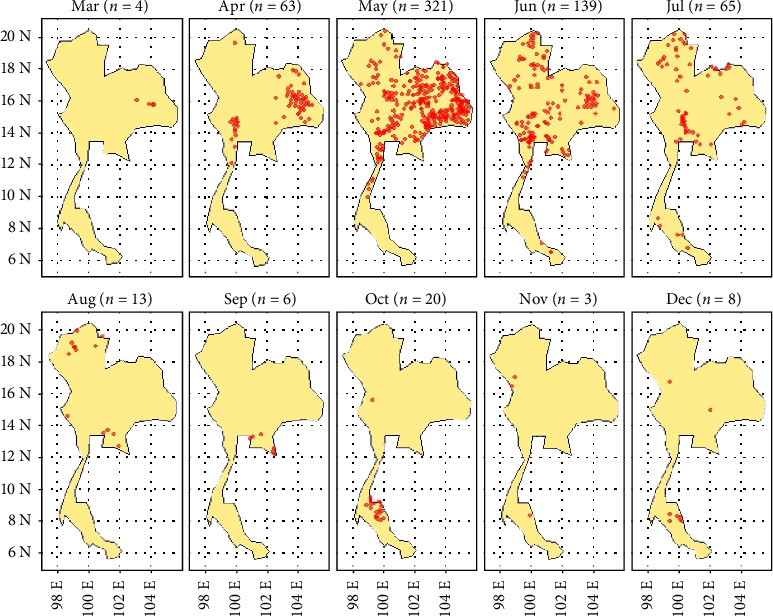
Temporal trend of the lumpy skin disease (LSD) outbreak in Thailand from March to December 2021. The red dot represents the LSD outbreak location, and *n* denotes the number of LSD outbreaks. The outbreak started in March and reached its peak in May, with a noticeable decrease observed after July.

During the period from March to December 2021, a total of 642 LSD cases were reported from a total of 928 districts. During this period, a total of 400,389 cases were reported across the country. The outbreak began in March and peaked in May. Subsequently, a noticeable decrease was observed after July. In April, numerous LSD outbreaks were concentrated in the northeastern regions, with some occurrences also noted in the north and west. By May, LSD outbreaks were widespread across the country, except in the southern region. Throughout June and July, LSD outbreaks were found in the northern, central, and northeastern regions. In the southern region, only a few LSD outbreaks were reported in July, October, November, and December (Figure 1).

### 3.2. Choropleth Map

**Figure 2:**
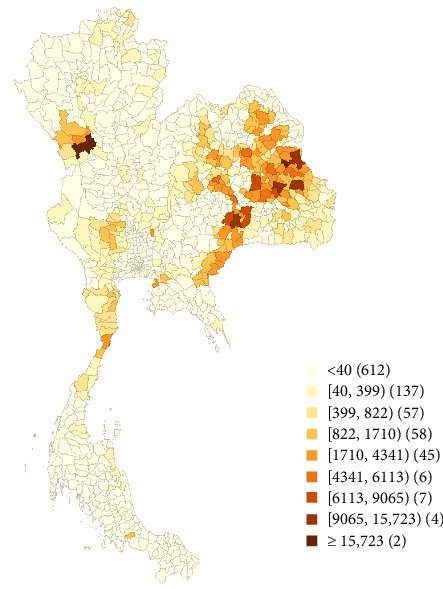
The total lumpy skin disease cases in districts of Thailand during 2021, grouped by nine Jenks natural breaks indicated by different colors. A distinct clustering pattern can be observed, primarily in the southeast region with many notable hotspots. However, there are fewer similar areas in the northern and southern regions.

The analysis revealed a noticeable clustering pattern, predominantly observed in the southeast region, characterized by several prominent hotspots. In contrast, fewer areas were evident in the northern and southern regions (Figure 2).

### 3.3. Spatial Autocorrelation on Moran's I

The LSD outbreak in Thailand during 2021 displayed a significant positive autocorrelation according to Moran's I analysis. The null hypothesis positing a random pattern of LSD outbreak distribution was consistently rejected, as indicated by a *p*-value <0.05. Moreover, the autocorrelation distribution of LSD outbreaks throughout 2021 revealed Moran's I coefficients, reaching 0.367, alongside corresponding *Z* values of 18.7612 (Figure S1).

**Figure 3:**
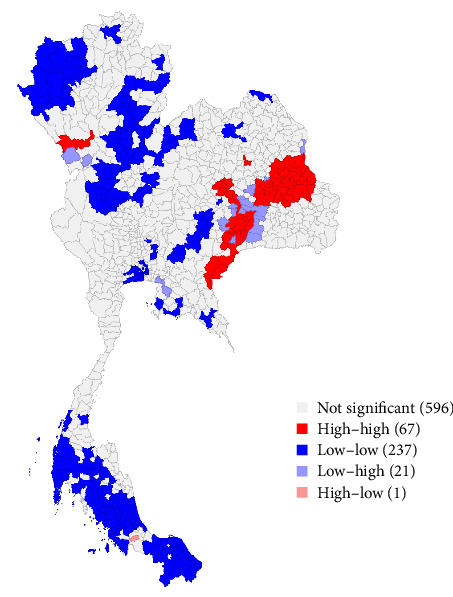
Local indicators of spatial association in a cluster map of lumpy skin disease outbreak spatial autocorrelation based on the number of cases in Thailand during 2021. The number of districts for each category is indicated in parentheses. The five patterns (e.g., high–high and low–low) are denoted by different colors. A distinct pattern in disease occurrence across various districts can be observed, with most showing either high–high or low–low occurrences clustered together. Some districts show either low occurrences surrounded by high occurrences or vice versa.

The geographical distribution of LSD outbreaks (Figure 3) provides a comprehensive narrative of the spatial patterns characterizing the outbreak. The results from LISA demonstrate the spatial diversity in LSD outbreak distributions in the region. The majority of districts exhibited clustering patterns indicative of high–high or low–low occurrences, reflecting localized variations in disease occurrence based on the number of cases. Intriguingly, outliers were identified, manifesting low–high and high–low spatial autocorrelation patterns, introducing nuanced complexities into the spatial dynamics under examination. Consistent with observations from the choropleth map, regions with high LSD cases surrounded by similar occurrences were primarily located in the eastern and northern regions of Thailand. On the other hand, areas with fewer cases of LSD were scattered across various geographical regions in Thailand. Notably, only one district (Khlong Hoi Kong) was in the high–low pattern category.

### 3.4. Directional Distribution of the LSD Outbreak

**Figure 4:**
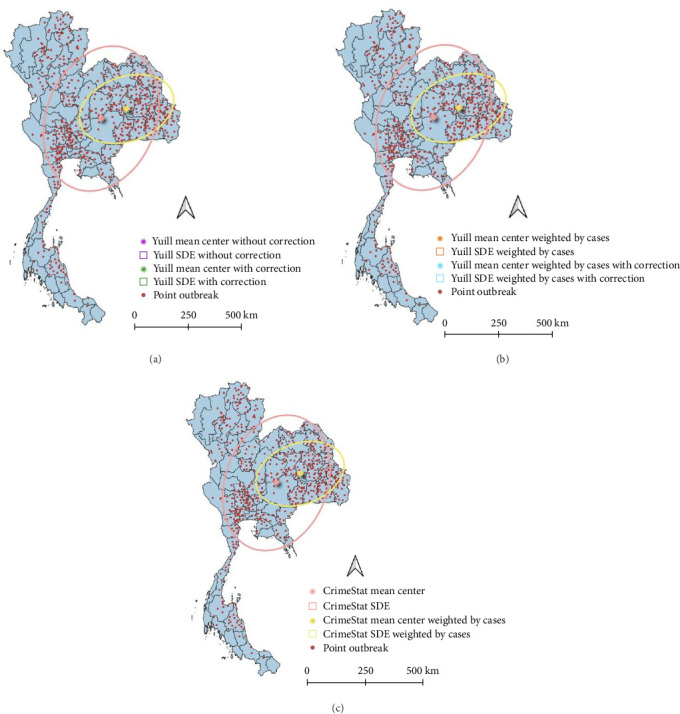
Comparison of the directional ellipse of the lumpy skin disease outbreak in Thailand, 2021, using Yuill and CrimeStat methods with unweighted and case-weighted settings. A star indicates the mean center, and a colored line ellipse. The ellipses and mean center produced by the Yuill method with corrections are identical to those generated by the CrimeStat method. (a) Unweighted standard deviational ellipses using the Yuill method; (b) Case-weighted standard deviational ellipses using the Yuill method; and (c) Unweighted and case-weighted standard deviational ellipses using the CrimeStat method.

The SDE spread pattern for LSD in Thailand was analyzed employing two different methods, Yuill and CrimeStat, two different settings used for the Yuill (Figure 4). Following the SDE analysis, where case weighting was implemented through the CrimeStat methodology, it was observed that the resulting outcomes closely corresponded to those obtained using the Yuill method with corrections (Table 1). However, upon conducting the analysis without weighting, discrepancies emerged primarily in the MajorSD and MinorSD parameters.

### 3.5. Spatio-Temporal Clusters

The comparative analysis of clusters derived from contrasting models and diverse MRCS configurations provided different results (Tables 2 and 3). Overall, the STP models generated more clusters than the Poisson, but most had a smaller spatial radius.

**Figure 5:**
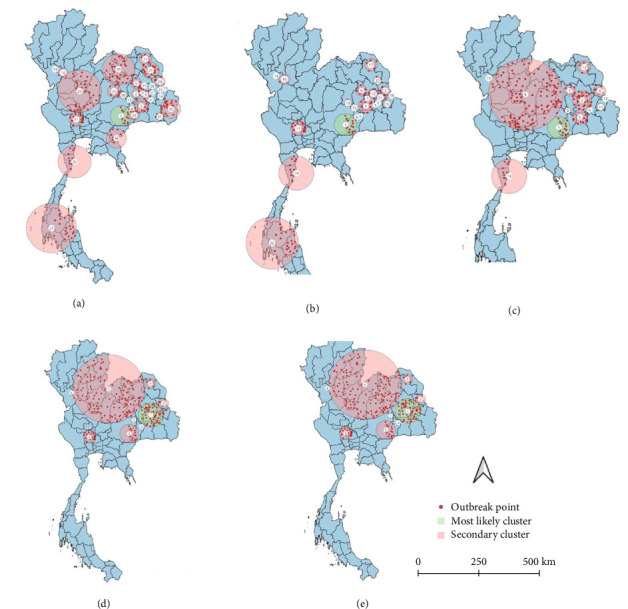
Clusters of lumpy skin disease outbreaks in Thailand during 2021, identified using the space–time permutation model with varying Maximum Report Cluster Sizes (MRCS), including (a) 5% MRCS, (b) 10% MRCS, (c) 15% MRCS, (d) 30% MRCS, and (e) 50% MRCS. The green circles denote the most likely clusters, while the pink circles indicate secondary clusters.

**Figure 6:**
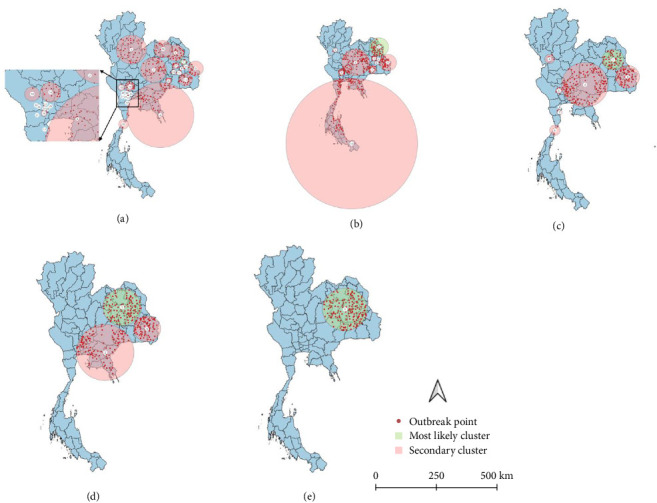
Lumpy skin disease outbreak clusters in Thailand during 2021, identified using the Poisson space–time model, with varying maximum report cluster sizes (MRCS), including (a) 5% MRCS, (b) 10% MRCS, (c) 15% MRCS, (d) 30% MRCS, and (e) 50% MRCS. Green circles indicate the most likely clusters, while pink circles represent secondary clusters.

All spatio-temporal models identified the primary cluster. The STP models identified 30 secondary clusters at a 5% MRCS, 21 secondary clusters at 10% MRCS, 13 secondary clusters at 15% MRCS, and 8 secondary clusters at both 30% and 50% MRCS (Figure 5). Meanwhile, the Poisson model identified 25 secondary clusters at a 5% MRCS, 8 secondary clusters at both 10% and 15% MRCS, and 2 secondary clusters at a 30% MRCS, with no secondary clusters detected at the 50% MRCS setting (Figure 6). As intuitively expected, the results also suggest that in small MRCS settings, the radius tends to be smaller.

## 4. Discussion

This study is the first to report the spatial epidemiology of the LSD outbreak in Thailand using nationwide data. The findings suggest that LSD outbreaks had spatial distribution patterns, a notable directional trend, and spatio-temporal patterns.

The temporal trend showed that the number of cases peaked in May, followed by a gradual reduction starting in June, decreasing substantially in July and subsequent months. Notably, this downturn in reported cases aligns with the initiation of a nationwide vaccination campaign by governmental authorities in June 2021 [47]. A previous study using interrupted time series analysis demonstrated that the implementation of nationwide vaccination in June 2021 associated with the reduction of LSD cases limiting the occurring new outbreak areas (Figure 1) [48]. Concurrently, additional control measures were also implemented, such as closing cattle markets, restricting cattle trading and movement, and controlling insect populations on farms, which contributed to the reduction in the number of outbreaks.

The choropleth map illustrates a pronounced number of LSD cases in the northeast, eastern, and southeastern regions, along with sporadic clusters observed in the north. Furthermore, based on the findings of this study, the districts harboring confirmed cases exhibit evident spatial autocorrelation, indicative of interconnectivity in the spread of LSD across geographical areas. Furthermore, according to the LISA results, the hotspot areas for LSD outbreaks were mostly located in the northeastern region. These findings align with the actual situation, where numerous LSD outbreaks were found in a district with a high number of LSD outbreak reports, surrounded by districts also experiencing a high number of LSD reports. It is reasonable to expect interconnectedness in the spread of LSD within neighboring areas, considering that insect vectors primarily contribute to short-distance transmission. Earlier research indicates the presence of vectors in various regions of Thailand, emerging as a contributing factor to LSD transmission [8].

The results from SDE provide detailed insight into the centers and directional patterns of the nationwide outbreak. Based on unweighted analysis, the epidemic appears to be spreading across Thailand from the northern to southern regions. However, when examining the ellipse weighted by cases, it becomes evident that the distribution trend originates from the northeast and extends toward the southwest. Furthermore, upon comparing the results of this study with a previous analysis of LSD outbreak data using SDE [10], the findings align with those of the earlier study, indicating a distribution trend extending from the north to the south and from the northeast to the southwest of Thailand. The first outbreak in Thailand is likely due to long-distance transmission. Given that the affected area was relatively far from the border, short-distance transmission by insect vectors appears less likely as the primary source. Moreover, a previous outbreak investigation in the affected area suggested that the LSD outbreak may have been introduced into Thailand through the illegal movement of infected animals from the source country. Alternatively, the legal movement of asymptomatic, LSD-carrying animals across the border could also be a factor, as infected animals may not display clinical signs. In addition, since the findings reveal the occurrence of LSD outbreaks in disparate areas, some being considerable distances apart (e.g., the distance between provinces with LSD outbreaks in the northeastern and northern regions exceeding 700 km), it is hypothesized that the pathway of animal movement may contribute to this transmission pattern as discussed in a previous study in Russia [49]. According to the aforementioned study, the potential transport of infected animals facilitated by vehicles was the major reason for LSDV spreading in Russia, with LSD cases observed at distances exceeding 800 km from the center of the outbreak, indicating long-distance transmission.

According to spatio-temporal analyses, the northeastern region of Thailand has become the focal point of the outbreak, with numerous primary and secondary clusters identified in this area. Nevertheless, numerous secondary clusters emerged throughout the nation with ranging radii. Furthermore, several districts showed autocorrelation within the clusters. Based on these findings, it is hypothesized that the long-distance spread could be attributed to animal movement from other outbreak areas, as evidenced by some most likely clusters having radii exceeding 100 km. The spread of LSD from the northeastern region to other areas, such as the western and southern regions, is also likely due to animal movements. Although animal movement controls were implemented following the first outbreaks, the disease may still spread through LSD-carrying animals that show no clinical signs or possibly through illegal animal movements. Additionally, the contamination of vehicles by insect vectors could further contribute to the spread of LSD across regions [49]. Conversely, in local outbreak areas (e.g., subdistricts and districts), the spread of LSD is likely due to insect vectors, which are commonly abundant in Thailand throughout the year [50]. Additionally, research conducted in Thailand using kernel transmission models demonstrates that the majority of herd-to-herd transmissions of LSD occur over short distances, with a median transmission estimated at between 0.2 and 0.6 km. These findings provide support for the speculation that LSDV is likely spread locally via insect vectors [51]. Previous research on the spatio-temporal patterns of LSD outbreaks in Thailand has primarily focused on specific regions like the northeast and north [6, 35], thereby restricting the comprehensive understanding of LSD epidemiology at the national level. This study addresses this gap by investigating the spatio-temporal patterns of LSD outbreaks using nationwide data. Therefore, the findings from this study contribute to a more comprehensive understanding, facilitating the development of prevention and control strategies at the national level. Furthermore, the results of this study align with the policy implemented by the livestock authority, which suggests administering an LSD vaccine to mitigate outbreaks in Thailand within a radius of 5–50 km from LSD outbreak centers in high-risk zones [47, 48, 52]. Moreover, in the present study, the STP model tends to generate more clusters with smaller radii compared to the Poisson model, aligning with the findings of previous smaller-scale studies [6]. This outcome is anticipated since these two methods utilize distinct data inputs and calculation methodologies.

This study examined the effects of different spatio-temporal parameter settings on the results, recognizing their significant impact on outcomes. It did not rely solely on the default setting since the results may include clusters that are too large, potentially diminishing their meaningfulness. Additionally, the present study varied MRCS rather than MSWS since the latter introduces statistical issues due to multiple comparisons [41]. In this study, it was observed that reducing the MRCS setting likely resulted in smaller cluster sizes. The findings also revealed that the number of clusters detected varied according to the model and MRCS setting. The different scenarios provided in this study may better accommodate the varying needs of users or authorities. For instance, if users aim for an overall exploration, a large value setting (e.g., 50% MRCS) will serve this purpose well since the resulting spatio-temporal clusters are likely to be large. However, a 50% MRCS setting could be inappropriate for investigating outbreak clusters in local areas since the resulting clusters would be too large, posing challenges in capturing local spatio-temporal patterns. If the objective is to detect local clusters, such as when the DLD authority targets an area with a specific radius (e.g. 25 km for insect vector controls), the use of a small MRCS setting would be appropriate for this purpose. Furthermore, given that each country has different geographical characteristics, density of cattle farms, proximity among farms, distinct policies, and varying levels of budget and resources, the determination of outbreak clusters should account for these components. Therefore, the analysis should not rely solely on a one-size-fits-all approach using default values, but rather investigate the most suitable models with advanced parameter settings.

This study is subject to certain limitations. The number of reported LSD cases might not reflect the actual count because some outbreaks may go unreported, often due to herd owners failing to report cases. Nevertheless, this limitation may not exert a substantial influence, given the active LSD outbreak detection by DLD authorities, as mentioned in several studies [6, 47, 50]. Furthermore, the cases were identified and reported based on the clinical signs observed in the animals. Laboratory confirmations were not conducted on samples from all farms, but rather from some animals on certain farms in the outbreak areas, aligning with common government practices and budget constraints. This limitation is widely acknowledged because, during large outbreaks, it is common practice to rely on clinical signs for reporting LSD cases due to constraints in time, cost, and resources, as mentioned in various studies [33, 35, 53, 54].

Ultimately, conducting a retrospective examination of spatial analysis on LSD occurrence can unveil crucial insight into the spatio-temporal dynamics of LSD distribution across time and space. The insight gained from this analysis serves as a fundamental resource for disease surveillance, control measures, and vector control strategies in Thailand.

## 5. Conclusion

This study represents the first effort to determine the burden of LSD outbreaks across Thailand by utilizing nationwide data. It also highlights the spatial and temporal patterns of disease outbreaks. The use of spatio-temporal analysis on animal disease significantly enhances the understanding of disease dynamics and provides stakeholders with a solid basis for developing causal hypotheses and implementing effective control measures.

## Figures and Tables

**Table 1:** The standard deviation ellipse characteristics derived from unweighted and weighted settings using both the Yuill and CrimeStat methods.

SDE	Center_X	Center_Y	MajorSD	MinorSD	Rotation angle (°)	Eccentrici
Unweighted Yuill	101.90	15.35	2.48	1.81	20.51	0.68
Unweighted Yuill with correction	101.90	15.35	2.48	1.81	20.51	0.68
Weighted Yuill	103.09	15.81	1.61	1.07	70.41	0.74
Weighted Yuill with correction	103.09	15.81	2.29	1.52	70.41	0.74
Unweighted CrimeStat	101.90	15.35	3.51	2.57	20.51	0.68
Weighted CrimeStat	103.09	15.81	2.29	1.52	70.41	0.74

*Note:* The data show the variations depending on whether the data are weighted and which calculation method is used.

**Table 2:** The number of spatio-temporal clusters of lumpy skin disease outbreaks detected using space–time permutation (STP) and Poisson models.

Model	MRCS (%)	Number of clusters	Average cluster size (km)
STP	5	31	36.97
STP	10	22	30.23
STP	15	14	43.09
STP	30	9	56.40
STP	50	9	56.40
Poisson	5	26	41.44
Poisson	10	8	169.90
Poisson	15	8	61.10
Poisson	30	3	148.57
Poisson	50	1	154.81

*Note:* The number of clusters produced varied across different models and maximum report cluster size (MRCS) settings. The STP model identified more clusters with a smaller radius compared to the Poisson model.

**Table 3:** The characteristics of the most likely clusters of lumpy skin disease outbreaks based on space–time permutation and Poisson models with various maximum reported cluster size settings.

Model	Coordinates/radius (km)	Time frame	Number of cases	Expected cases	Relative risk	LLR^a^	*p*-Value
STP 5% MRCS	(14.694110 N, 102.527350 E)/61.59	22 May 2021–28 May 2021	13,705	1126.99	—	21,860.25	<0.001
STP 10% MRCS	(14.694110 N, 102.527350 E)/62.72	22 May 2021–28 May 2021	15,850	1250.42	—	25,924.89	<0.001
STP 15% MRCS	(14.694110 N, 102.527350 E)/62.72	22 May 2021–28 May 2021	15,850	1250.42	—	25,924.89	<0.001
STP 30% MRCS	(15.842370 N, 103.864256 E)/82.87	6 March 2021–16 Apr 2021	59,224	19,145	—	28,987.26	<0.001
STP 50% MRCS	(15.842370 N, 103.864256 E)/82.87	6 March 2021–16 Apr 2021	59,224	19,145	—	28,987.26	<0.001
Poisson 5% MRCS	(16.450689 N, 104.333282 E)/27.12	8 May 2021–14 May 2021	40,182	112.25	397.81	198,296.01	<0.001
Poisson 10% MRCS	(17.612614 N, 104.496298 E)/151.15	1 May 2021–21 May 2021	76,020	1144.35	81.77	251,625.77	<0.001
Poisson 15% MRCS	(16.703237 N, 103.844180 E)/84.86	3 Apr 2021–11 June 2021	115,745	8583.91	18.56	210,165.91	<0.001
Poisson 30% MRCS	(16.699740 N, 103.154900 E)/138.75	3 Apr 2021–11 June 2021	176,879	22,000.86	13.61	251,029.70	<0.001
Poisson 50% MRCS	(16.284453 N, 103.267377 E)/154.81	3 Apr 2021–11 June 2021	260,638	38,311.58	17.64	366,745.81	<0.001

^a^LLR = log likelihood ratio.

## Data Availability

The data used in this study are available from the World Animal Health Information System (WAHIS) at https://wahis.woah.org.

## Supporting Information

Supporting Information Figure S1. Moran's I analysis of lumpy skin disease (LSD) outbreak of Thailand year 2021: (a) final statistic of LSD outbreak of Thailand; (b) Moran scatter plot of LSD outbreak spatial autocorrelation in Thailand.
