# Supplementary figures and images for: Geographical Distribution, Spatial Directional Trends, and Spatio-Temporal Clusters of the First Rapid and Widespread Lumpy Skin Disease Outbreaks in Thailand
Source: Transbound Emerg Dis. 2025 Feb 24;2025:4900775. doi: 10.1155/tbed/4900775 (PMC12016731; doi:10.1155/tbed/4900775)

a) permutation: 999  
pseudo p-value: 0.001000

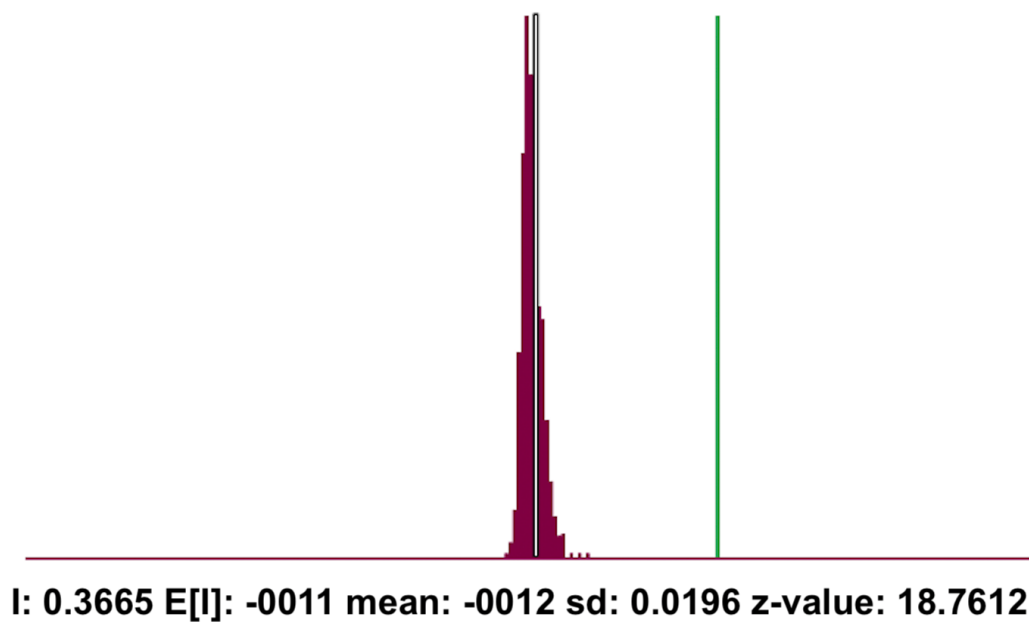

b) **Moran's I: 0.367 (isolates in weights are removed)**

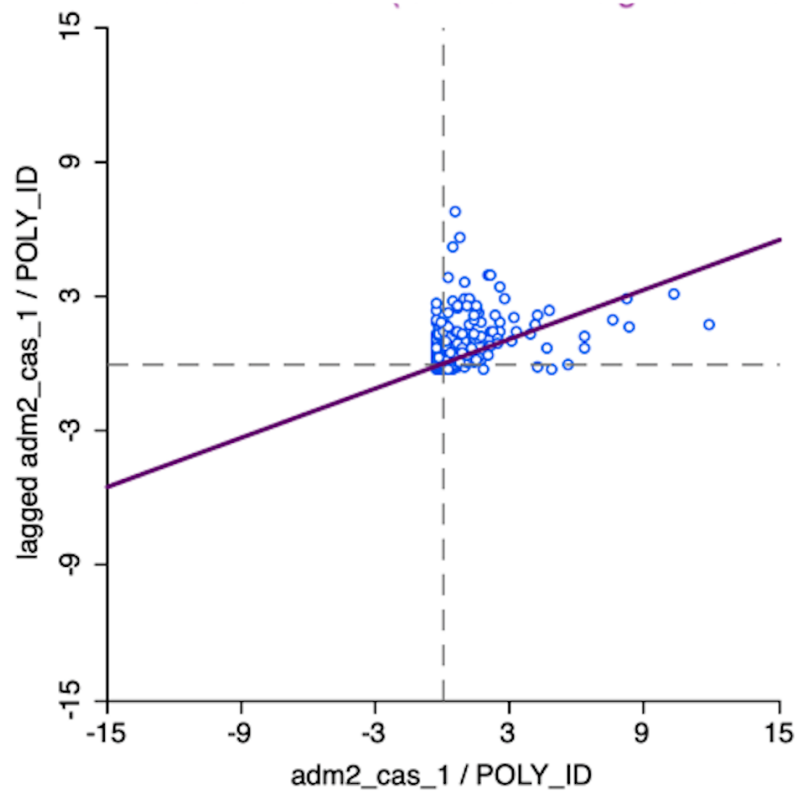

Supplement: Supporting Information — Figure S1. Moran's I analysis of lumpy skin disease (LSD) outbreak of Thailand year 2021: (a) final statistic of LSD outbreak of Thailand; (b) Moran scatter plot of LSD outbreak spatial autocorrelation in Thailand. [file 4900775.f1.pdf]
